# Supplementary material for: Analysis of PPARγ Signaling Activity in Psoriasis
Source: Int J Mol Sci. 2021 Aug 10;22(16):8603. doi: 10.3390/ijms22168603 (PMC8395241; doi:10.3390/ijms22168603)
Supplement: Supplementary file 1 [file ijms-22-08603-s001.zip › Supplemental materials_Analysis of PPARg signaling activity in psoriasis/Pathway models/Models images and html files/Differentiation of psoriatic T cells/6592.html]

JAK1


# Protein JAK1

|  |  |
| --- | --- |
| URN | urn:agi-llid:3716 |
| Total Entities | 0 |
| Connectivity | 11657 |
| Name | JAK1 |
| Description | Janus kinase 1 |
| Notes | This gene encodes a membrane protein that is a member of a class of protein-tyrosine kinases (PTK) characterized by the presence of a second phosphotransferase-related domain immediately N-terminal to the PTK domain. The encoded kinase phosphorylates STAT proteins (signal transducers and activators of transcription) and plays a key role in interferon-alpha/beta and interferon-gamma signal transduction. Alternative splicing results in multiple transcript variants. [provided by RefSeq, Mar 2016] |
| Primary Cell Localization | Cytoplasm |
| Class | Protein kinase |
| ObjectType | Protein |

---

|  |  |
| --- | --- |
| Pathway | I-cell: CCK-8 Secretion and Regulation of Eating Behavior |
|  | T-Cell Dependent B-Cell Activation |
|  | V(D)J Recombination Activation |
|  | Immunoglobulin Class-Switch Recombination Activation |
|  | Immunoglobulin Genes Transcriptional Activation |
|  | T-Cell Maturation (Hypothesis) |
|  | Natural Killer Cell Activation |
|  | CD8+ T-Cell Activation |
|  | T-Cell Cytotoxic Mediated Cell Death |
|  | Th1-Cell Differentiation |
|  | Treg-Cell Differentiation |
|  | Th2-Cell Differentiation |
|  | AHR Signaling in Treg and Dendritic Cells Function |
|  | AHR Signaling in Th17 Cells Function |
|  | Th17-Cell Differentiation |
|  | AHR Signaling in Tr1 Cells Function |
|  | CXC Chemokine Receptor Signaling |
|  | CC Chemokine Receptor Signaling |
|  | Plasmin Effects in Inflammation |
|  | Proplatelet Maturation |
|  | AHR in Intestinal Cell Antimicrobial Barrier Maintenance |
|  | Basophil Activation |
|  | Eosinophil Survival by Cytokine Signaling |
|  | Mast-Cell Activation without Degranulation through CXCR4 Signaling |
|  | Mast-Cell Activation without Degranulation |
|  | Mast-Cells De Novo Synthesized Mediators via IgE Independent Signaling |
|  | Macrophage M1 Lineage |
|  | CD16/CD14 Proinflammatory Monocyte Activation |
|  | AHR Signaling in M1 Macrophages Function |
|  | anti-Inflammatory Function of Macrophage M2 Lineage |
|  | AXL Receptor Inhibits Macrophages and Dendritic Cells Function |
|  | Neutrophil Recruitment and Priming |
|  | Negative Acute Phase Proteins Synthesis |
|  | Positive Acute Phase Proteins Synthesis |
|  | Vascular Endothelial Cell Activation by Cytokines |
|  | Adipokines Production by Adipocyte |
|  | Nociception Expression Targets Signaling |
|  | CCR1 Expression Targets |
|  | IL6 Expression Targets -> Nociception |
|  | Proteins Involved in Atherosclerosis |
|  | Th1-Cells Activation and Proliferation in Atherosclerosis |
|  | Cardiomyocyte Hypertrophy |
|  | Hypertrophic Cardiomyopathy |
|  | TRPM4/6/7/8 Signaling in Arterial Hypertension (Hypothesis) |
|  | Preconditioning Ischemia |
|  | Lymphocyte Mediated Myocardial Injury in Myocarditis |
|  | SOCS in Negative Feedback Regulation in Myocarditis |
|  | Hair Follicle Keratinocyte Apoptosis |
|  | Acute Phase in Atopic Dermatitis |
|  | Atopic Dermatitis |
|  | Melanoma |
|  | Apoptosis Block in Melanoma Cell |
|  | T-Cells Differentiation Block in Psoriasis |
|  | IFN-gamma/TNF-alpha Mediated Cell Proliferation in Psoriasis |
|  | IL22 Induces Keratinocyte Proliferation in Psoriasis |
|  | Dendritic Cell Activation in Systemic Lupus Erythematosis |
|  | Th17-Cell Function in Systemic Lupus Erythematosus |
|  | Th1-Cell Function in Systemic Lupus Erythematosus |
|  | B-Cell Activation in Systemic Lupus Erythematosus |
|  | Th2-Cell Function in Systemic Lupus Erythematosus |
|  | Oxidative Stress in Vitiligo |
|  | Acute Cytotoxic CD8+T-Cell Response against Melanocytes |
|  | Vitiligo |
|  | T-Cell Cytotoxic Response against Melanocytes in Vitiligo |
|  | Humoral Immunity in Vitiligo |
|  | Treg-Cell Function in Diabetes Mellitus Type 1 |
|  | Natural Killer T-Cell Roles in Diabetes Mellitus Type 1 |
|  | non-Suppressive Treg-Cell in Diabetes Mellitus Type 1 |
|  | TLR2 Signaling in Treg-Cell in Type 1 Diabetes (Animal Model) |
|  | Natural Killer Cell in Diabetes Mellitus Type 1 |
|  | Toll-like Receptors in beta-Cell |
|  | Peripheral Tolerance to Autoantigens Recession in Diabetes Mellitus Type 1 |
|  | Treg-Cell Activation in Diabetes Mellitus |
|  | Donohue Syndrome Progression (Hypothesis) |
|  | Tubular Cell Dysfunction in Progressive Diabetic Nephropathy |
|  | Endothelial Cell Dysfunction in Progressive Diabetic Nephropathy |
|  | Proteins Involved in Diabetic Nephropathy |
|  | Proliferative Diabetic Retinopathy |
|  | Adipokines and Cytokines in Insulin Resistance in Skeletal Muscles |
|  | Proteins Involved in Insulin Resistance |
|  | Adiponectin Synthesis Declines in Insulin Resistance |
|  | Insulin Resistance in Hepatocytes |
|  | Oxidation of Fatty Acid in non-Alcoholic Fatty Liver Disease |
|  | beta-Cell Destruction through Cytokines in Diabetes Mellitus |
|  | beta-Cell Destruction in Diabetes Mellitus Type 1 |
|  | Hashimoto's Thyroiditis |
|  | Thyroid Hormones in Adipose Tissue Metabolism |
|  | Thyroid Dysfunction |
|  | Graves Ophthalmopathy |
|  | Immune System Activation in Hyperthyroidism |
|  | Thyroid Stimulating Hormone (TSH) Resistance in Congenital Hypothyroidism |
|  | Overt Hypothyroidism, Primary |
|  | Thyrotropin Releasing Hormone (TRH) Hypothalamic Release in non-Autoimmune Hypothyroidism |
|  | Central Hypothyroidism |
|  | Tertiary Hypothyroidism |
|  | Adipokines Production by Adipocyte Impaired in Obesity |
|  | CD4+T-Cell Response in Celiac Disease |
|  | CD8+ T-Cell Response in Celiac Disease |
|  | Metastatic Colorectal Cancer |
|  | Leukocyte Migration toward the Endothelial Cell in Ulcerative Colitis |
|  | Th2-Cell Function in Ulcerative Colitis |
|  | FLT3/KIT/MLL Signaling in Leukemia |
|  | Apoptosis Block in Acute Myeloid Leukemia Cell |
|  | RARA Signaling in Acute Myeloid Leukemia (M3) |
|  | Acute Myeloid Leukemia |
|  | B-Cell Acute Lymphoblastic Leukemia |
|  | Diffuse Large-B-Cell Lymphoma |
|  | Diffuse Large-B-Cell Lymphoma ABC Subtype |
|  | Primary Mediastinal B-cell Lymphoma (PMBL) Subtype |
|  | Follicular Lymphoma |
|  | Hodgkin Lymphoma |
|  | Hodgkin and Reed-Sternberg Cells Reprogramming |
|  | Mantle Cell Lymphoma |
|  | IL6/IGF1/VEGFA Signaling in Multiple Myeloma |
|  | Multiple Myeloma |
|  | T-Cell Acute Lymphoblastic Leukemia |
|  | Hepatocellular Carcinoma |
|  | Growth Factor Signaling in Hepatocellular Carcinoma |
|  | Pancreatic Ductal Carcinoma |
|  | Growth Factor Signaling in Pancreatic Neoplasms |
|  | TRPM7/8 in Pancreatic Adenocarcinoma |
|  | Eosinophil Survival in Asthma |
|  | TSLP Signaling in Bronchial Epithelial Cell in Asthma |
|  | Periostin (POSTN) Production by Airway Epithelium in Asthma |
|  | Airway Smooth Muscle Cell High Rate Proliferation |
|  | Goblet-Cell Exocytosis in Asthma |
|  | Goblet-Cell Related Mucus Secretion in Asthma |
|  | Club-Cell Role in Asthma |
|  | Basophil Activation in Asthma |
|  | B-Cell Mediated IgE Production in Asthma |
|  | Th17-Cell Differentiation in Asthma |
|  | Th2-Cell Response in Asthma |
|  | Mucin Production in Goblet Airway Epithelial Cells |
|  | Osteoclast Activation in Gout |
|  | Neutrophil Recruitment in Sinovium in Gout |
|  | Dendritic Cell Dysfunction in Psoriatic Arthritis |
|  | Th17-Cell and Th1 Immune Responsein Psoriatic Arthritis |
|  | Osteoclast Activation in Psoriatic Arthritis |
|  | Keratinocyte Activation in Psoriatic Arthritis |
|  | Synovial Fibroblast Proliferation in Rheumatoid Arthritis |
|  | Osteoclast Activation in Rheumatoid Arthritis |
|  | B-Cells Function in Systemic Scleroderma |
|  | Th2-Cells Function in Systemic Scleroderma |
|  | Skin Fibrosis |
|  | CCR5 Signaling in Macrophages |
|  | Th17-Cell Numbers Reduction in HIV |
|  | CD4+ T-Cell Function Decline in HIV |
|  | CD4+ T-Cell Death in HIV |
|  | Diabetes Induced Periodontitis |
|  | Cowden Syndrome |
|  | Gluten Impact on Neuronal System (Hypothesis) |
|  | Bone Loss in Osteoporosis |
|  | BDNF Upregulation Triggered by Seizures |
|  | Glioma Invasion Signaling |
|  | Glioma Stem Cell Program Activation |
|  | IL6/IL12 Signaling Activates Immune System in Multiple Sclerosis |
|  | ERBB/VEGFR/Akt Signaling in Breast Cancer |
|  | IGF1R/AKT Signaling in Breast Cancer |
|  | ESR1/ERBB Positive Luminal Breast Cancer |
|  | Breast Cancer |
|  | ESR1 Signaling in Breast Cancer |
|  | Endometrial Cancer |
|  | Endometrioid Endometrial Cancer |
|  | Clear Cell Endometrial Cancer and Papillary Serous Endometrial Cancer |
|  | Endometrioid Ovarian Carcinoma |
|  | Clear Cell Ovarian Carcinoma |
|  | High-Grade Serous Ovarian Carcinoma |
|  | Low-Grade Serous Ovarian Carcinoma |
|  | Ovarian Cancer |
|  | Mucinous Ovarian Carcinoma |
|  | IL6/JAK/STAT3 Signaling in Cholesteatoma |
|  | Cholesteatoma |
|  | Prostate Cancer |
|  | IL10/STAT3 Signaling in M2 Macrophage and Retinal Angiogenesis |
|  | Neurotrophic Factor Deprivation in Retinal Ganglion Cell Death |
|  | Cytokines Trigger Otitis Media |
|  | Alveolar Macrophage Negative Regulation Declined in COPD |
|  | Mucin Hyperproduction in Goblet and Mucous Cells |
|  | Endothelial Cell Dysfunction in Glomerulonephritis |
|  | Endothelial Cell Dysfunction in Pyelonephritis |
|  | IFNG -> ARRB1/STAT1 Signaling |
|  | CLCF1 Expression Targets |
|  | CTF1 Expression Targets |
|  | CXCL12 Expression Targets |
|  | CCL3 Expression Targets |
|  | CCL3L1 Expression Target |
|  | CCL16 Expression Targets |
|  | CCL5 Expression Targets |
|  | CCL7 Expression Target |
|  | CCL7 Expression Targets |
|  | CCL8 Expression Targets |
|  | CCL2 Expression Targets |
|  | CSF3 Expression Targets |
|  | CSF2 -> STAT Expression Targets |
|  | LIF Expression Targets |
|  | CNTF Expression Targets |
|  | Clozapine Induced Granulocytopenia |
|  | GH2/GHR Expression Target |
|  | GH1/GHR -> STAT Expression Targets |
|  | CSH1/GHR Expression Targets |
|  | Receptors and Adaptor Proteins Activated in Cancer |
|  | Genes with Mutations in Cancer-Associated Sustaining of Proliferative Signaling |
|  | STAT3 and NFkB Activate Inflammation-Induced Tumorigenesis |
|  | IL23A and IL17A Provoke Cancer-Associated Inflammation |
|  | STAT3 Inhibits Maturation of Dendritic Cells and Facilitates Cancer Progression |
|  | STAT3 Facilitates the Function of Treg Cells and Cancer Progression |
|  | IL13/STAT6/ZEB1 Signalling Promotes Cancer Metastatic Progression |
|  | N2 Neutrophils in Tumor-Promoting Inflammation and Tumor Progression |
|  | VEGFA Dependent Angiogenesis in Cancer |
|  | Epithelial to Mesenchymal Transition in Cancer: Overview |
|  | Cancer Cells Inhibit Adipocyte Differentiation |
|  | Skeletal Muscle Wasting in Cancer Cachexia |
|  | Treg Cells Promote Immunosuppression in Cancer Immune Escape |
|  | Myeloid Derived Suppressor Cells in Cancer Immune Escape |
|  | IDO1 in Cancer Immune Escape |
|  | IGF2 -> STAT Expression Targets |
|  | IGF1 -> STAT Expression Targets |
|  | Insulin -> STAT Expression Targets |
|  | IGF1R -> STAT Signaling |
|  | IFNLR1-> STAT/NFKB Signaling |
|  | IFNW1/IFNR Expression Target |
|  | IFNA1/IFNR Expression Targets |
|  | IFNB1/IFNR Expression Targets |
|  | IFNG/IFNR Expression Targets |
|  | IL22R -> STAT3 Signaling |
|  | IL13R -> STAT6 Signaling |
|  | IL31R -> STAT Signaling |
|  | IL15R -> STAT Signaling |
|  | IL7R -> FOXO/NF-kB Signaling |
|  | IL15R -> NF-kB/NFATC Signaling |
|  | IL2R -> ELK/SRF/MYC Signaling |
|  | IL10R -> STAT Signaling |
|  | IL13R -> STAT Signaling |
|  | IL3R -> STAT Signaling |
|  | IL27R -> STAT Signaling |
|  | IL7R -> STAT Signaling |
|  | IL9R -> STAT Signaling |
|  | IL5R -> STAT Signaling |
|  | IL11R -> STAT3 Signaling |
|  | IL6R -> STAT Signaling |
|  | IL2R -> STAT Signaling |
|  | IL6ST -> STAT5B Signaling |
|  | IL4R -> STAT Signaling |
|  | IL23R -> STAT3/NF-kB Signaling |
|  | IL21R -> STAT Signaling |
|  | IL4R -> ELK/SRF/HMGY Signaling |
|  | IL7 Expression Targets |
|  | IL13 Expression Targets |
|  | IL21 Expression Targets |
|  | IL15 Expression Targets |
|  | IL3 Expression Targets |
|  | IL6 Expression Targets |
|  | IL10 Expression Targets |
|  | IL5 Expression Targets |
|  | IL22 Expression Targets |
|  | IL11 Expression Targets |
|  | IL9 Expression Targets |
|  | IL31 Expression Targets |
|  | IL4 Expression Targets |
|  | IL2 Expression Targets |
|  | UrokinaseR -> ELK/SRF Signaling |
|  | UrokinaseR -> STAT Signaling |
|  | PTPRC -> STAT6 Expression Targets |
|  | OSM/OSMR Expression Targets |
|  | OSM/IL6ST/LIFR Expression Targets |
|  | PRL/GHR -> STAT Expression Targets |
|  | PTPRC -> STAT6 Signaling |
|  | FOXO1 Signaling |
|  | Ras-GAP Regulation Signaling |
|  | LTA Expression Targets |
|  | TNF -> STAT Expression Targets |
|  | TNFRSF1A -> STAT Signaling |
|  | B-Cell Differentiation Inhibition by Dioxin |
|  | PLAU -> STAT1 Expression Targets |
|  | PLAU -> ELK-SRF/AP-1 Expression Targets |
|  | PPAR Psoriasis |
|  | pprarg neg, uknown targets, ps-positive |
|  | Model of PPARG signaling in psoriasis |
|  | New Pathway (3) |
|  | dsRNA induces innate immunity and INFs in alveolar cells |
|  | IFN signaling in alveolar cells |
|  | Dendritic Cell Activation in COVID19 |
|  | 2\_Acute phase initiation in atopic dermatitis |
|  | 1\_Differentiation of psoriatic T cells |
|  | 2\_Interleukin-17 and interleukin-22 signaling in psoriasis |
|  | 2\_The role of endothelial cells in pyelonephritis |
|  | 3\_Role of neurotrophic factor deprivation in retinal ganglion cell death |
|  | 2\_Cardiomyocyte hypertrophy |
|  | 3\_2\_Regulation of airway surface liquid (ASL) thickness |
|  | 3\_1\_Mucus accumulation in asthma |
|  | 2\_2\_Eosinophilia and neutrophilia in asthma: eosinophil apoptosis |
|  | 4\_Mucin hyperproduction in goblet and mucous cells in COPD |
|  | 3\_Leukocyte migration toward the endothelial cells in the intestine microvasculature |
|  | 2\_Intestine epithelial cells drive immunological responses in ulcerative colitis |
|  | 2\_CD8+ T-cell response and mucosal damage in celiac disease |
|  | 5\_Tax-mediated dysregulation of NF-kB signaling in HTLV-1 infection |
|  | 1\_Inflammatory mediators increase the production of hepcidin (HAMP) |
|  | 4\_Inflammatory mediators inhibit GATA signaling in erythropoiesis |
|  | 4\_2\_Insulin resistance: inflammation-related insulin resistance |
|  | 4\_3\_Insulin resistance: FFA-related insulin resistance |
|  | 1\_Antigen-presenting cells promote islet dysfunction and immune system activation in T1D |
|  | 3\_Islet beta-cell destruction in T1D |
|  | 2\_Defective tolerance of autoreactive T-cell in T1D |
|  | 1\_Decreased secretion of thyroid hormones |
|  | Differentiation of psoriatic T cells |
|  | Interleukin-17 and interleukin-22 signaling in psoriasis |

---

|  |  |
| --- | --- |
| Group | Genes with Mutations Associated with B-Cell Acute Lymphoblastic Leukemia |
|  | Proteins Involved in B-Cell Acute Lymphoblastic Leukemia |
|  | Genes with Mutations Associated with T-Cell Acute Lymphoblastic Leukemia |
|  | Proteins Involved in Cowden Syndrome |

---

|  |  |
| --- | --- |
| MedScan ID | 3716 |

---

|  |  |
| --- | --- |
| LocusLink ID | 3716 |
|  | 16451 |
|  | 84598 |
|  | 230508 |
|  | 100022 |
|  | 319959 |
|  | 362552 |

---

|  |  |
| --- | --- |
| Alias | OTTMUSP00000008566 |
|  | BAP004 |
|  | Janus protein tyrosine kinase 1 |
|  | Janus kinase I |
|  | JAK1B |
|  | Tyrosine-protein kinase JAK1 |
|  | MGC37919 |
|  | JAK1A |
|  | LOC105378775 |
|  | AA960307 |
|  | kinase JAK1 |
|  | Janus kinase 1 |
|  | Jak I |
|  | C130039L05 |
|  | Tyrosine-protein kinase JAK I |
|  | C130039L05Rik |
|  | Janus kinase 1 (a protein tyrosine kinase) |
|  | JAK1 |
|  | JTK3 |
|  | LOC362552 |
|  | RP23-336G7.2 |
|  | Janus protein tyrosine kinase I |
|  | JAK1 kinase |
|  | kinase JAK I |
|  | RIKEN cDNA C130039L05 gene |
|  | OTTHUMP00000010896 |
|  | JAK-1 |
|  | expressed sequence AA960307 |

---

|  |  |
| --- | --- |
| EC Number | 2.7.10.2 |
|  | 2.7.10.1 |
|  | 2.7.1.112 |

---

|  |  |
| --- | --- |
| GO ID | 0005524 |
|  | 0031730 |
|  | 0005131 |
|  | 0046872 |
|  | 0004715 |
|  | 0019903 |
|  | 0004713 |
|  | 0031625 |
|  | 0000165 |
|  | 0019221 |
|  | 0060333 |
|  | 0035722 |
|  | 0035723 |
|  | 0038110 |
|  | 0038114 |
|  | 0070106 |
|  | 0070757 |
|  | 0070102 |
|  | 0038111 |
|  | 0038113 |
|  | 0035556 |
|  | 0018108 |
|  | 1903672 |
|  | 0006468 |
|  | 0060334 |
|  | 0046677 |
|  | 0060337 |
|  | 0005737 |
|  | 0005856 |
|  | 0005829 |
|  | 0005768 |
|  | 0005925 |
|  | 0016020 |
|  | 0005634 |
|  | 0016301 |
|  | 0000166 |
|  | 0004672 |
|  | 0016740 |
|  | 0036016 |
|  | 0098761 |
|  | 0007167 |
|  | 0016310 |
|  | 1904894 |
|  | 0007260 |
|  | 0005088 |
|  | 0005622 |
|  | 0045087 |
|  | 0031234 |
|  | 0016477 |
|  | 0043547 |
|  | 0007243 |
|  | 0007169 |
|  | 0046777 |
|  | 0005102 |
|  | 0005515 |
|  | 0016772 |
|  | 0038083 |
|  | 0012505 |
|  | 0030154 |
|  | 0060338 |
|  | 0042127 |
|  | 0004718 |
|  | 0023034 |
|  | 0016021 |
|  | 0004872 |
|  | 0007242 |

---

|  |  |
| --- | --- |
| KEGG ID | hsa:3716 |
|  | rno:84598 |
|  | mmu:16451 |

---

|  |  |
| --- | --- |
| MedLine Reference | 10967130 |
|  | 12087100 |
|  | 12477932 |
|  | 12487370 |
|  | 12584205 |
|  | 15126250 |
|  | 16413512 |
|  | 10200816 |
|  | 10349636 |
|  | 10374907 |
|  | 10526570 |
|  | 10781830 |
|  | 10872802 |
|  | 10922068 |
|  | 11042159 |
|  | 11058581 |
|  | 11076861 |
|  | 11114734 |
|  | 11217851 |
|  | 11390995 |
|  | 11437454 |
|  | 12466851 |
|  | 12576323 |
|  | 12738762 |
|  | 15254900 |
|  | 15456882 |
|  | 15659653 |
|  | 15782199 |
|  | 1581631 |
|  | 15923602 |
|  | 15988755 |
|  | 16141072 |
|  | 16141073 |
|  | 16280321 |
|  | 16417589 |
|  | 16737695 |
|  | 17114464 |
|  | 1848670 |
|  | 2466296 |
|  | 2482828 |
|  | 7490523 |
|  | 7539296 |
|  | 7542761 |
|  | 7544789 |
|  | 7579387 |
|  | 7589562 |
|  | 7613027 |
|  | 7713924 |
|  | 7796811 |
|  | 7929391 |
|  | 8331382 |
|  | 8514334 |
|  | 8535092 |
|  | 8593242 |
|  | 8608597 |
|  | 8662243 |
|  | 8889548 |
|  | 8918879 |
|  | 8934548 |
|  | 9229117 |
|  | 9342212 |
|  | 9360952 |
|  | 9524251 |
|  | 9548458 |
|  | 9553136 |
|  | 9590172 |
|  | 9638363 |
|  | 9889194 |
|  | 10433356 |
|  | 10502458 |
|  | 10531356 |
|  | 10586060 |
|  | 10702271 |
|  | 10825200 |
|  | 10875931 |
|  | 10899310 |
|  | 10982844 |
|  | 10993906 |
|  | 11016959 |
|  | 11133764 |
|  | 11162588 |
|  | 11163768 |
|  | 11201744 |
|  | 11418623 |
|  | 11468294 |
|  | 11527382 |
|  | 11557047 |
|  | 11722592 |
|  | 11742534 |
|  | 11786531 |
|  | 11801527 |
|  | 12130510 |
|  | 1213395 |
|  | 12133952 |
|  | 12200137 |
|  | 12223098 |
|  | 12374810 |
|  | 12551917 |
|  | 12559972 |
|  | 12620806 |
|  | 12777975 |
|  | 12960323 |
|  | 1373877 |
|  | 15883164 |
|  | 15894543 |
|  | 16102578 |
|  | 16239216 |
|  | 16767694 |
|  | 7499365 |
|  | 7512720 |
|  | 7698020 |
|  | 7973659 |
|  | 8232552 |
|  | 8272872 |
|  | 8536716 |
|  | 8700888 |
|  | 8702790 |
|  | 8756628 |
|  | 8995399 |
|  | 9020188 |
|  | 9178903 |
|  | 9417082 |
|  | 9446616 |
|  | 9452495 |
|  | 9492017 |
|  | 9516124 |
|  | 9566874 |
|  | 9774657 |
|  | 15194700 |
|  | 17158029 |
|  | 17433443 |
|  | 8247543 |
|  | 8022486 |
|  | 11244571 |

---

|  |  |
| --- | --- |
| Organism | Homo sapiens {Organism urn:agi-taxid:9606} |
|  | Mus musculus {Organism urn:agi-taxid:10090} |
|  | Rattus norvegicus {Organism urn:agi-taxid:10116} |
|  | Homo sapiens |
|  | Mus musculus |
|  | Rattus norvegicus |

---

|  |  |
| --- | --- |
| Mouse chromosome position | 4 46.19 cM |
|  | 4 46.3 cM |

---

|  |  |
| --- | --- |
| OMIM ID | 147795 |

---

|  |  |
| --- | --- |
| Rat chromosome position | 5q33 |
|  | 5q31.3-q35 |

---

|  |  |
| --- | --- |
| Hugo ID | 6190 |
|  | HGNC:6190 |

---

|  |  |
| --- | --- |
| Human chromosome position | 1p31.3 |
|  | 1p32.3-p31.3 |

---

|  |  |
| --- | --- |
| Swiss-Prot Accession | P23458 |
|  | Q6P669 |
|  | P23458.2 |
|  | B1ASP2 |
|  | Q3U8P8 |
|  | Q3URU8 |
|  | P52332.1 |
|  | G3V9W2 |
|  | Q59GQ2 |
|  | Q9UD26 |
|  | P52332 |
|  | Q0VGA4 |
|  | Q4LDX3 |
|  | Q8IV82 |
|  | Q3TI10 |
|  | Q3UZ30 |
|  | Q8CFX4 |
|  | Q8K0I7 |
|  | O35803 |
|  | Q5FVF5 |
|  | Q8CIZ4 |
|  | JAK1\_HUMAN |
|  | B1ASP2\_MOUSE |
|  | Q3U8P8\_MOUSE |
|  | Q6ZRA6 |

---

|  |  |
| --- | --- |
| PIR ID | A39577 |

---

|  |  |
| --- | --- |
| GenBank ID | NC\_000001 |
|  | NM\_001321856 |
|  | NP\_001308785 |
|  | NM\_001320923 |
|  | NP\_001307852 |
|  | NM\_001321852 |
|  | NP\_001308781 |
|  | NM\_001321854 |
|  | NP\_001308783 |
|  | NM\_001321853 |
|  | NP\_001308782 |
|  | NM\_001321857 |
|  | NP\_001308786 |
|  | NM\_002227 |
|  | NP\_002218 |
|  | NM\_001321855 |
|  | NP\_001308784 |
|  | NG\_023402 |
|  | AC093427 |
|  | AL357078 |
|  | AL590436 |
|  | AL606517 |
|  | CH471059 |
|  | EAX06547 |
|  | EAX06548 |
|  | HC002094 |
|  | CBG22571 |
|  | KC876867 |
|  | KC876881 |
|  | KF495968 |
|  | KT584867 |
|  | AB209057 |
|  | BAD92294 |
|  | AB219242 |
|  | BAE02826 |
|  | AK128375 |
|  | AK314257 |
|  | BC009079 |
|  | BC023604 |
|  | AAH23604 |
|  | BC062431 |
|  | AAH62431 |
|  | BC111401 |
|  | AAI11402 |
|  | BC132729 |
|  | AAI32730 |
|  | BX647675 |
|  | BX648044 |
|  | HQ628631 |
|  | AER93381 |
|  | M64174 |
|  | AAA36527 |
|  | P23458 |
|  | NC\_000070 |
|  | NM\_146145 |
|  | NP\_666257 |
|  | XM\_006502812 |
|  | XP\_006502875 |
|  | XM\_011240451 |
|  | XP\_011238753 |
|  | NM\_013567 |
|  | AL627251 |
|  | AL645668 |
|  | CH466527 |
|  | EDL30874 |
|  | AK029232 |
|  | AK033004 |
|  | AK081552 |
|  | AK133866 |
|  | AK134143 |
|  | BAE22031 |
|  | AK141210 |
|  | BAE24589 |
|  | AK143650 |
|  | AK148521 |
|  | AK152128 |
|  | BAE30969 |
|  | AK161759 |
|  | AK168058 |
|  | BAE40036 |
|  | AK180982 |
|  | AK181063 |
|  | AK181487 |
|  | AK183753 |
|  | AK196334 |
|  | AK208150 |
|  | BC024100 |
|  | BC031297 |
|  | AAH31297 |
|  | BC039752 |
|  | AAH39752 |
|  | BC058993 |
|  | M33425 |
|  | AAA40016 |
|  | S63728 |
|  | AAB27517 |
|  | P52332 |
|  | NC\_005104 |
|  | XM\_006238452 |
|  | XP\_006238514 |
|  | XM\_006238453 |
|  | XP\_006238515 |
|  | NM\_053466 |
|  | NP\_445918 |
|  | AC\_000073 |
|  | AABR07049385 |
|  | AAHX01038760 |
|  | CH473998 |
|  | EDL97824 |
|  | AF540911 |
|  | AAN59910 |
|  | AJ000556 |
|  | CAA04187 |
|  | BC090029 |
|  | AAH90029 |
|  | FQ227839 |
|  | FQ231507 |
|  | FQ233794 |
|  | XM\_006502813 |
|  | CAM21388 |
|  | CAM21389 |
|  | XP\_005270898 |
|  | AMYH02001172 |
|  | AMYH02001173 |
|  | AABR06038934 |
|  | XP\_011540812 |
|  | AABR06038935 |
|  | XM\_011549220 |
|  | XP\_011539697 |
|  | AC\_000026 |
|  | ABBA01074552 |
|  | ABBA01074553 |
|  | ABBA01074554 |
|  | XP\_006502876 |
|  | XP\_006710687 |
|  | XM\_005270841 |
|  | XP\_011547522 |
|  | AC\_000133 |
|  | NC\_018912 |
|  | XM\_011542510 |
|  | XM\_006710624 |
|  | XM\_011541395 |
|  | AAHY01038557 |
|  | AAHY01038558 |
|  | NT\_032977 |
|  | NW\_001838579 |
|  | NW\_921351 |
|  | AC\_000044 |
|  | Q0VGA4 |
|  | Q4LDX3 |
|  | Q6P669 |
|  | Q8IV82 |
|  | NT\_039264 |
|  | NW\_001030747 |
|  | Q3TI10 |
|  | Q3U8P8 |
|  | Q3URU8 |
|  | Q3UZ30 |
|  | Q8CFX4 |
|  | Q8K0I7 |
|  | NW\_047717 |
|  | NW\_001084842 |
|  | O35803 |
|  | Q5FVF5 |
|  | Q8CIZ4 |
|  | Q9UD26 |
|  | XM\_342872 |
|  | XP\_342873 |
|  | XM\_001061647 |
|  | XP\_001061647 |
|  | BAC87406 |
|  | Q59GQ2 |
|  | Q6ZRA6 |
|  | BG067874 |
|  | BG074678 |
|  | BF224937 |
|  | BG080932 |
|  | BG087301 |
|  | NW\_000211 |
|  | BQ032637 |
|  | 2288925 |
|  | 24415844 |
|  | 58476629 |
|  | 81866487 |
|  | 81886638 |
|  | 81909629 |
|  | 123783325 |
|  | 123788792 |
|  | 123794305 |
|  | 123795003 |
|  | 1708580 |
|  | 200576 |
|  | 21410529 |
|  | 27370733 |
|  | 74137337 |
|  | 74189929 |
|  | 74207575 |
|  | 74212448 |
|  | 81878055 |
|  | 81878341 |
|  | 8248854 |
|  | AA960307 |
|  | 111494091 |
|  | 119626952 |
|  | 121946632 |
|  | 121948500 |
|  | 124375862 |
|  | 125060 |
|  | 190735 |
|  | 27469796 |
|  | 34535713 |
|  | 38382952 |
|  | 62087694 |
|  | 68299593 |
|  | 74711103 |
|  | 74720332 |
|  | 74728049 |
|  | 74737464 |
|  | 74741308 |
|  | 119626953 |
|  | 123254037 |
|  | 148698927 |
|  | 149044565 |

---

|  |  |
| --- | --- |
| Swiss-Prot ID | JAK1\_HUMAN |
|  | B1ASP2\_MOUSE |
|  | Q3URU8\_MOUSE |
|  | Q3U8P8\_MOUSE |

---

|  |  |
| --- | --- |
| Cell Localization | Endomembrane system |
|  | Cytoplasm |

---

|  |  |
| --- | --- |
| Ensembl ID | ENSG00000162434 |
|  | ENSP00000343204.4 |
|  | ENST00000342505.5 |
|  | ENSMUSG00000028530 |
|  | ENSMUSP00000099842.3 |
|  | ENSMUST00000102781.9 |
|  | ENSRNOG00000011157 |
|  | ENSRNOP00000061649.1 |
|  | ENSRNOT00000064542.1 |
|  | ENST00000342505.4 |
|  | ENSMUSP00000099842 |
|  | ENSMUST00000102781 |
|  | ENSRNOT00000064542 |
|  | ENST00000342505 |
|  | ENSRNOP00000061649 |
|  | ENSP00000343204 |

---

|  |  |
| --- | --- |
| MGI ID | MGI:96628 |
|  | 96628 |
|  | 2140180 |
|  | 2443062 |

---

|  |  |
| --- | --- |
| RGD ID | 69056 |

---

|  |  |
| --- | --- |
| Unigene ID | Hs.207538 |
|  | Mm.289657 |
|  | Rn.90191 |
|  | Mm.185636 |

---

|  |  |
| --- | --- |
| Homologene ID | 1678 |

---

|  |  |
| --- | --- |
| Shape | Sickle-vertex |

---

|  |  |
| --- | --- |
| IPI ID | IPI00784013 |
|  | IPI00775993 |
|  | IPI00126078 |
|  | IPI00649788 |
|  | IPI00212981 |

---

|  |  |
| --- | --- |
| Homo sapiens Chromosome position | 1p32.3-p31.3 |

---

|  |  |
| --- | --- |
| Ensembl Transcript ID | ENST00000342505 |

---

|  |  |
| --- | --- |
| FunctionalClass | protein binding |
|  | protein-tyrosine kinase |
|  | ATP binding |
|  | Janus kinase |
|  | kinase |
|  | nucleotide binding |
|  | protein kinase |
|  | transferase |
|  | growth hormone receptor binding |
|  | receptor |

---

|  |  |
| --- | --- |
| Mus musculus Chromosome position | 4 46.3 cM |
|  | 4 |

---

|  |  |
| --- | --- |
| KEGG pathway | Adipocytokine signaling pathway |
|  | Jak-STAT signaling pathway |

---

|  |  |
| --- | --- |
| Rattus norvegicus Chromosome position | 5q31.3-q35 |
|  | 5q33 |

---

|  |  |
| --- | --- |
| Ensembl Gene ID | ENSG00000162434 |

---
